# Immunohistochemical Expression of Nuclear β-Catenin and STAT-6 in a Solitary Fibrous Tumor of the Soft Palate: A Case Report and Review of the Literature

**Published:** 2017-07-01

**Authors:** Ivonne Montes-Mojarro, Javier Baquera-Heredia, Juan Felipe Sanchez-Marle, Carlos Ortiz-Hidalgo

**Affiliations:** 1 *Surgical Pathology Department, The American British Cowdray Medical Center, Mexico city 01120, Mexico*; 2 *Head and Neck Surgery Department, The American British Cowdray Medical Center, Mexico city 01120, Mexico*

**Keywords:** Solitary Fibrous Tumor, Immunohistochemistry, Mesenchymal Tumors, Soft Palate, STAT-6, β-Catenin

## Abstract

The solitary fibrous tumors (SFT) are rare tumors in the head and neck region and there have been only 5 cases reported in the literature in the soft palate. The current paper presents a unique case of a 62-year-old male with TFS arising in the soft palate. The tumor was highly cellular, composed of bland looking haphazardly arranged spindle cells. The signal transducer and activator of transcription (STAT)-6 and nuclear β-catenin were reactive by immunohistochemistry (IHC). The current case highlights the importance of the STAT-6 and the β-catenin as IHC markers to make a differential diagnosis with other entities. In summary, the paper presents the first reported case of a SFT of the soft palate in a male patient with nuclear expression of STAT-6 and β-catenin.

## Introduction

Originally described in the pleural cavity 1931 by Klemperer and Rabin as a spindle cell neoplasia, solitary fibrous tumor (SFT) is an uncommon, CD34 positive fibroblastic neoplasia described in virtually any anatomic location (1). SFT are rare tumors in the head and neck region, and are reported in the meninges, orbit, nose, paranasal sinuses, nasopharynx, parapharyngeal space, all major salivary glands, epiglottis, larynx, thyroid, skin, and deep soft tissues of the neck (2). To the best of the authors` knowledge, in the English language literature, 50 cases are reported in the oral cavity, and only 5 cases in the soft palate, and all of them in females (Table 1 (3, 4, 5, 6). The infrequent localization of the tumor in the soft palate remarks the challenge of the differential diagnosis, especially with hemangiopericytoma, sinonasal type; an entity with similar histopathologic features (7). The current paper describes herein the first case of a SFT of the soft palate in a male, with immunohistochemical expression of nuclear β-catenin and STAT-6.

## Case report

A 62-year-old male, presented with a 2-year history of dysphonia, nasal voice, and dyspnea. No history of prior surgery or trauma affecting the region was reported. On physical examination, a painless, mobile, non-ulcerated mass was detected in the soft palate that was excised. Macroscopically, the tumor weighed 9.1 g, was ovoid and well demarcated, and measured 3.2x3x2.4 cm. The outer surface was smooth and showed a focal linear pedicle with 2.7 cm in length.

Histologically, the lesion was well circumscribed with a thick fibrous capsule. The tumor was highly cellular, composed of bland looking haphazardly arranged spindle cells with an oval nuclei, inconspicuous nucleoli and scant eosinophilic cytoplasm. There was no necrosis and mitosis. There were few bands of collagen fibers between cells and numerous irregular staghorn-type variably dilated and branching thin-walled vessels. Few scattered mast cells were identified. Immunohistochemistry (IHC) was performed (see the list of primary antibodies in Table 1)**. **Positive immunoreactivity was demonstrated in the tumor cells for CD34, Bcl-2, factor XIIIa, cytoplasmic CD99, focal reactivity for epithelial membrane antigen (EMA), and strong signal transducer and activator of transcription (STAT)-6 and β-catenin nuclear staining pattern. Ki 67 index was 5%. The neoplastic cells were uniformly negative for D240, S-100 protein, cytokeratin AE1-AE3, actin (HHF35), transducin-like enhancer protein (TLE)-1, and activin receptor-like kinase (ALK)-1. Based on the histological and immunohistochemical findings, a diagnosis of solitary fibrous tumor was rendered (Table 2).

**Figure 1:**
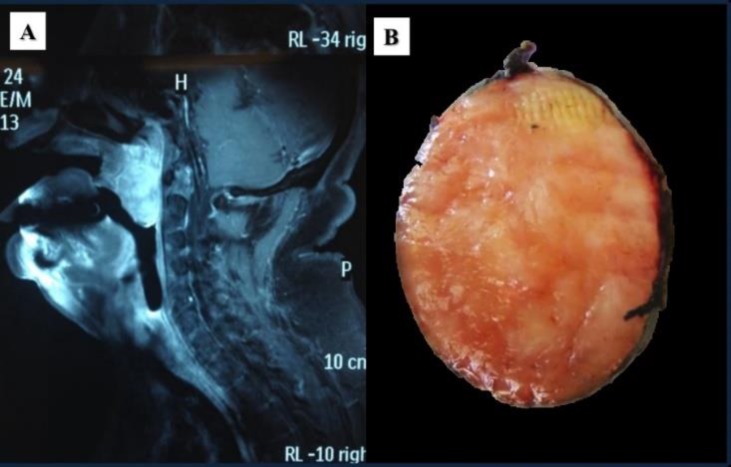
A) Gadolinium-enhanced, sagittal T1-weighted image showing a mass located in the soft palate. B) An ovoid, encapsulated mass measured 3.2x3x2.4 cm. The cut section was firm with a yellow-red surface.

**Figure 2:**
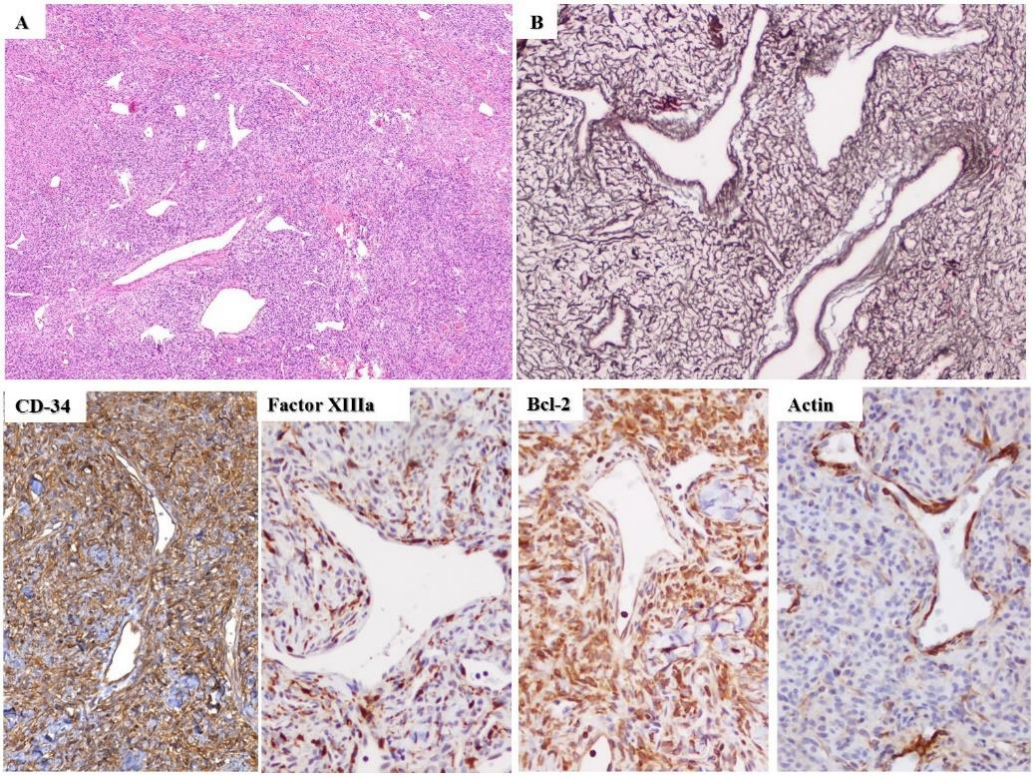
A) Solitary fibrous tumor composed of bland, spindled-shaped cells in a patternless pattern, with variably dense, intervening fibrous stroma and numerous irregular staghorn-type, variably dilated and branching thin-walled vessels. B) Gordon-Sweet reticulin staining; Lower panel: tumor cells showing labeling for CD34, factor XIIIa, Bcl-2, and actin.

**Fig 3:**
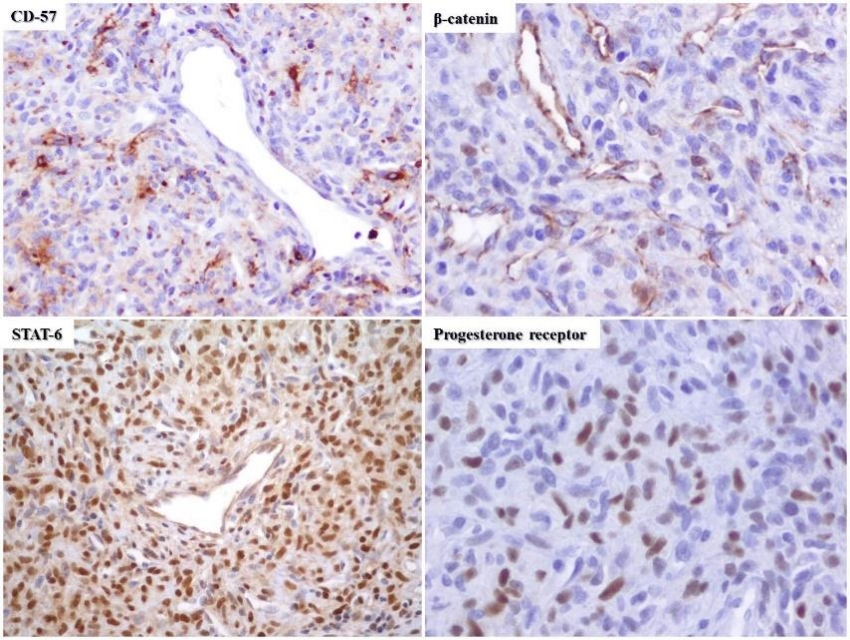
Tumor cells demonstrating nuclear expression of β-catenin, STAT-6, progesterone receptors, and granular cytoplasmic positivity for CD57.

**Table 1:** Primary Antibodies and Their Sources, Clones, and Dilutions Used in the Present Study

Antibody	Clone	Source	Dilution
CD34	QBend10	Bio SB	1:100
Bcl-2	124	DAKO/USA	1:50
Factor XIIIa	Factor XIIIa	BioCare	1:200
CD99	12E7	DAKO/USA	1:50
EMA	E29	DAKO/USA	1:200
STAT-6	Polyclonal	Biotechnology	1:200
ᵦ-Catenin	Monoclonal	Biotechnology	1:1000
Ki-67	EP5	Bio SB	1:50
S-100	Polyclonal Rabbit	DAKO/USA	1:2000
AE1-AE3	AE1-AE3	Bio SB	1:100
TLE-1	IF5	Bio SB	1:30
ALK-1	ALK-1	DAKO/USA	1:30
Progesterone receptor	PgR	DAKO/USA	1:50
Actin	HHF35	Bio SB	1:25
CD57	NK1	Bio SB	1:100
D240	D2410	DAKO	1:100

EMA, epithelial membrane antigen; STAT-6, signal transducer and activator of transcription-6; TLE-1, transducin-like enhancer protein-1; ALK-1, Anaplastic lymphoma kinase 1

## Discussion

Solitary fibrous tumor can be found at any location of the body with more frequent appearance at the pleura, lower extremities or the retroperitoneum, but rarely within the oral cavity (2). Fewer than 40 cases with SFT were reported in the oral cavity, the buccal mucosa is the most frequent site of occurrence, followed by the tongue and lower lip (2). The soft palate location is extremely uncommon with only 5 cases described in the English language literature, and all of them in females (3, 4, 5 y 6). The current study reported the 1st case of a SFT affecting the soft palate in a 62-year-old male (Table 2).

Intraoral SFT is presented as a slow-growing asymptomatic of varying size. STF of soft tissues and pleura are associated with fever, chest pains, and hypoglycemia, but none of these features is reported in patients with intraoral SFT tumors (1). In all the previous reports, the tumors presented a slow growing mass without systemic symptoms involvement; in the current case there was a similar clinical presentation. 

Due to the fascicular proliferation and the prominent branching hemangiopericytoma like vasculature, histologically, SFT may mimic other tumors such as cellular angiofibroma, desmoid fibromatosis, dermatofibrosarcoma protuberans, gastrointestinal stromal tumor, fibromyxoid sarcoma, malignant peripheral nerve sheath tumor, synovial sarcoma, sarcomatoid mesothelioma, schwannoma, soft tissue perineurioma, and spindle cell lipoma (1,7). Another important diagnosis that is necessary to review is the hemangiopericytoma, sinonasal type; this entity has the same histological appearance and then the immunohistochemical is crucial to make the differential diagnosis (Table 3). However, immunohistochemical studies may differentiate SFT form its mimics. SFT shows reactivity for CD34 (80%-90%), Bcl-2 (30%), factor XIIIa and CD99 (70%), nuclear β-catenin (40%), and EMA (20%-30%). CD34 is the best sensible marker to diagnose SFT; however, new immunohistochemical markers such as STAT-6 and β-catenin have been recently identified (6). Recently, a highly recurrent fusion on chromosome 12q13 spanning the loci of NAB2 and STAT6 is described (9 y 10). The role in tumorigenesis is attributed to STAT-6 in various malignancies (9). It is postulated that *NAB2/STAT-6* fusion leads to a nuclear relocation of STAT-6. Therefore, STAT-6 IHC is proposed as a reliable surrogate marker for *NAB2/STAT-6* fusion. Doyle et al., demonstrated that STAT-6 immunohistochemistry has the specificity to dismiss tumors with absent fusions, which are histologically similar. The current case showed strong diffuse nuclear reactivity for STAT-6 (9).

**Table 2:** History of Solitary Fibrous Tumors in the Soft Palate

Genger / Age (year)	Symptoms	Diagnosis	Immunohistochemistry	Treatment / Follow-up Without Recurrence	References
Female / 60	Pharyngeal foreign body	SFT	CD34 + / Bcl-2 +	Resection / 20 months	3
Female /27	No symptoms	SFT	CD34 + / Vimentin + / Bcl-2 + / CD99 + / SMA + / S-100 +	Resection /16 months	3
Female / 35	Pharyngeal foreign body	SFT	Vimentin, CD34 + / Bcl-2 -	Resection / 15 years	4
Female / 80	Slow growing mass	SFT	Vimentin, CD34+ / Bcl-2 +	Resection / 10 years	5
Female / 50	Moveable mass in soft palate	SFT	CD34 +	Resection / ?	6
Male / 62	Dysphonia, nasal voice, and dyspnea	SFT	CD34 + / Bcl-2 + / Factor XIIIa + / CD99 + / EMA + / STAT-6 + / β-catenin +	Resection / ?	The present case

SFT, Solitary fibrous tumor **EMA, **epithelial membrane antigen; STAT-6, signal transducer and activator of transcription-6

**Table 3:** Immunochemistry in Differential Diagnosis of Solitary Fibrous Tumors

Diagnosis	CD34	D240	Bcl-2	Factor XIIIa	CK AE1/3	CD31	S-100	SMA
SFT	+ (100%)	-*	+ (95%)	+ (40%)	-	-	-	-
Neurofibroma	+ (48%)	-	+ (43%)	-	-	-	+ (92%)	-/+
Myofibroma	-	-	-	+ (27%)	+ (31%)	-	-	+ (49%)
Benign fibrous histyocioma	-	+	-	+ (52%)	-	-	-	+ (36%)
Synovial sarcoma monophasic	+ (2%)	-	+ (94%)	-	+ (63%)	-	+ (27%)	+ (15%)
Hemangiopericytoma “sinonasal type”.	+ (8%)	+*	+ (2%)	+ (78%)	-	-	+ (4%)	+ (81%)
Diagnosis	**CD99**	**CD57**	**Rb**	**PR**	**ᵦ** **-catenin (nuclear)**	**TLE-1**	**EMA**	**STAT-6**
SFT	+ (70%)	+ (42%)	+ (79%)	+	+ (40%)	+ (12%)	+ (3%)	+ (100%)
Neurofibroma	-	+ (94%)	N/A	+ (75%)	-	-	-	-
Myofibroma	-	-	+ (11%)	N/A	+ (8%)	-	+ (4%)	-
Benign fibrous histyocioma	-	-	N/A	N/A	N/A	N/A	-	-
Synovial sarcoma monophasic	+ (63%)	+ (27%)	N/A	N/A	+ (55%)	+ (90%)	+ (73%)	-
Hemangiopericytoma “sinonasal type”.	N/A	N/A	N/A	N/A	N/A	N/A	-	N/A

SFT: Solitary fibrous tumor; N/A: Not available:

* Ref (13)

β-Catenin is a cytoplasmic protein normally located adjacent to the cytoplasmic membranes, where it interacts with cytoplasmic domains of transmembrane E-cadherin protein. It has 2 functions: To stabilize E-cadherin, and act as a transcriptional factor in the WNT (wingless integrated -1) signaling pathway. Mutation in the proteins involved with WNT signaling and β-catenin lead to the expression persistence of the protein, which promotes the translocation to the nucleus, activating genes associated with cell proliferation, and inhibition of apoptosis (12).

The prognosis of these neoplasms in general is based on the location, size, and histologic features such as hypercellularity and mitosis (>4 mitosis per 10 HPF). Recurrence or metastasis develops in 5% -10% of patients^7^. The pleural SFT presents a higher index (10%-20%) of malignancy; however, most of the extrapleural tumors are benign and cured by resection. When the metastasis occurs, it appears in the lung, bone, and liver. Although clinical follow-up of patients with oral SFT is limited, to date all of the intraoral tumors are treated by complete local excision and no additional treatment is necessary for any of the patients (2).

The elected treatment is surgery resection, with no adjuvant therapy, on head and neck tumors; the recurrence is rarely reported (2).

In summary, the current paper presented the first reported case of a SFT of the soft palate in a male patient with nuclear expression of STAT-6 and β-catenin.
